# A 17-molecule set as a predictor of complete response to neoadjuvant chemotherapy with docetaxel, cisplatin, and 5-fluorouracil in esophageal cancer

**DOI:** 10.1371/journal.pone.0188098

**Published:** 2017-11-14

**Authors:** Hajime Fujishima, Shoichi Fumoto, Tomotaka Shibata, Kohei Nishiki, Yoshiyuki Tsukamoto, Tsuyoshi Etoh, Masatsugu Moriyama, Norio Shiraishi, Masafumi Inomata

**Affiliations:** 1 Department of Gastroenterological and Pediatric Surgery, Oita University Faculty of Medicine, Yufu, Oita, Japan; 2 Department of Surgery, Oita Nakamura Hospital, Yufu, Oita, Japan; 3 Department of Molecular Pathology, Oita University Faculty of Medicine, Yufu, Oita, Japan; 4 Comprehensive Surgery for Community Medicine, Oita University Faculty of Medicine, Yufu, Oita, Japan; University of South Alabama Mitchell Cancer Institute, UNITED STATES

## Abstract

**Background:**

Recently, neoadjuvant chemotherapy with docetaxel/cisplatin/5-fluorouracil (NAC-DCF) was identified as a novel strong regimen with a high rate of pathological complete response (pCR) in advanced esophageal cancer in Japan. Predicting pCR will contribute to the therapeutic strategy and the prevention of surgical invasion. However, a predictor of pCR after NAC-DCF has not yet been developed. The aim of this study was to identify a novel predictor of pCR in locally advanced esophageal cancer treated with NAC-DCF.

**Patients and methods:**

A total of 32 patients who received NAC-DCF followed by esophagectomy between June 2013 and March 2016 were enrolled in this study. We divided the patients into the following 2 groups: pCR group (9 cases) and non-pCR group (23 cases), and compared gene expressions between these groups using DNA microarray data and KeyMolnet. Subsequently, a validation study of candidate molecular expression was performed in 7 additional cases.

**Results:**

Seventeen molecules, including transcription factor E2F, T-cell-specific transcription factor, Src (known as “proto-oncogene tyrosine-protein kinase of sarcoma”), interferon regulatory factor 1, thymidylate synthase, cyclin B, cyclin-dependent kinase (CDK) 4, CDK, caspase-1, vitamin D receptor, histone deacetylase, MAPK/ERK kinase, bcl-2-associated X protein, runt-related transcription factor 1, PR domain zinc finger protein 1, platelet-derived growth factor receptor, and interleukin 1, were identified as candidate molecules. The molecules were mainly associated with pathways, such as transcriptional regulation by SMAD, RB/E2F, and STAT. The validation study indicated that 12 of the 17 molecules (71%) matched the trends of molecular expression.

**Conclusions:**

A 17-molecule set that predicts pCR after NAC-DCF for locally advanced esophageal cancer was identified.

## Introduction

Esophageal cancer is a malignant tumor with a poor prognosis. It is considered that esophagectomy is the main strategy for esophageal cancer; however, the survival benefit with surgery alone is unsatisfactory, and the survival rates have been reported to be about 20%–30% at 2 years [1] and 10%–20% at 5 years [2]. In Western countries, neoadjuvant chemoradiotherapy is a standard treatment for resectable esophageal cancer [3–5]. In Japan, a randomized phase III trial (JCOG9204) showed that disease-free survival with adjuvant chemotherapy (AC) involving cisplatin/5-fluorouracil (5-FU) therapy (CF) was superior to that with surgery alone. In addition, a subsequent randomized phase III trial (JCOG9907) mentioned that the prognostic benefit of neoadjuvant chemotherapy (NAC) involving CF (NAC-CF) was superior to that of AC involving CF [6]. Accordingly, NAC-CF has become the standard preoperative treatment for locally advanced esophageal cancer in Japan. However, the survival benefit of NAC-CF is unsatisfactory. Recently, docetaxel/cisplatin/5-FU therapy (DCF) was presented as a novel strong regimen for esophageal cancer, and several studies have reported the safety of NAC involving DCF (NAC-DCF) [7, 8]. Studies have reported that the pathological complete response (pCR) rates of NAC-DCF and NAC-CF in locally advanced esophageal cancer were 12%–18% and 4%–6%, respectively [7–9]. Accordingly, NAC-DCF is a powerful regimen and is expected to achieve pCR in locally advanced esophageal cancer. Moreover, it was indicated that the prognoses of pCR cases with NAC-DCF for esophageal cancer were satisfactory [10, 11]. The prediction of pCR will have a great impact on the therapeutic strategy, such as avoidance of surgical treatment. However, biomarkers for clinical application in the prediction of pCR with NAC-DCF for esophageal cancer have not been identified.

Responsiveness to chemotherapeutic agents has been reported to be partly associated with genetic variations in pharmacokinetic and pharmacodynamic action [12]. The molecular background of regulating therapeutic effectiveness in esophageal cancer remains largely unclear. Some molecular markers have been reported for the tailored treatment of esophageal cancer, such as cisplatin-related markers (interferon-induced transmembrane protein 1 [*IFITM1*], breast cancer susceptibility gene 1 [*BRCA1*], and kallikrein-related peptidase 10 [KLK-10]) [13–15], 5-FU-related markers (thymidylate synthase [TSase] and dihydropyrimidine dehydrogenase [DPD]) [16–18], and docetaxel-related markers (*BRCA1* and identified beta 1 integrin [*ITGB1*]) [14, 19]. It has been reported that the serum p53 antibody might be a predictor of pathological tumor response to NAC-DCF in esophageal cancer [20]. However, these single molecular markers are still not used for clinical application. An intricate mechanism of drug sensitivity is the most difficult obstacle for the prediction of therapeutic efficacy [13]. Multiple factors are involved in drug response mechanisms. Additionally, key determinants of the response significantly vary among individuals, and the factors intricately interact. The multifactorial mechanisms limit the prediction of an individual drug response with any single marker [21–23]. We considered that the use of many molecules was more appropriate to predict pCR than the use of a single molecular biomarker. In fact, we have reported an 80-gene set to predict the response to NAC-radiotherapy (RT) in rectal cancer [24]. We considered exploring a gene set to predict pCR after NAC-DCF for esophageal cancer in this study.

The aim of this study was to identify the predictors of pCR after NAC-DCF for locally advanced esophageal cancer. We investigated gene expressions in clinical esophageal cancer samples and performed comparisons between pCR cases and non-pCR cases using DNA microarray data and KeyMolnet.

## Materials and methods

### Patients and human tissue samples

This cohort study included esophageal cancer patients treated with NAC-DCF followed by surgery at Oita University Hospital between June 2013 and March 2016. Thirty-two patients met the following inclusion criteria: (i) histological diagnosis of primary esophageal squamous cell carcinoma; (ii) stage IB/II/III according to UICC 7th edition; (iii) age ≤ 80 years; (iv) performance status of 0–1; and (v) no previous chemotherapy, thoracic RT, or thoracic surgery.

Esophageal cancer tissue samples were collected at biopsy during endoscopic examination before the administration of the first course of chemotherapy. The biopsy specimen was collected from an elevated part at the proximal side of the tumor in a unified manner. The specimens were frozen and preserved in a freezer maintained at −80°C. This study was approved by the Ethics Committee of Oita University Faculty of Medicine, and all patients included in this study provided written informed consent.

### Therapy

The NAC-DCF regimen consisted of a 1-h i.v. infusion of docetaxel (70 mg/m^2^) on day 1, a 2-h infusion of cisplatin (70 mg/m^2^) on day 1, and continuous i.v. infusion of 5-FU (750 mg/m^2^) on days 1–5. This regimen was administered every 3 weeks, and 3 scheduled courses were administered before esophagectomy. Surgery was scheduled to be carried out within 4–6 weeks after the last day of preoperative chemotherapy, when curative resection was considered possible.

### Response evaluation

The pathological response was evaluated according to the Japanese Classification of Esophageal Cancer 11th edition as follows: grade 0, no recognizable cytological or histological therapeutic effect; grade 1a, viable cancer cells account for two-thirds or more of the tumor tissue; grade 1b, viable cancer cells account for between one-third and two-thirds of the tumor tissue; grade 2, viable cancer cells account for less than one-third of the tumor tissue; grade 3, no viable cancer cells are apparent (pCR) [25, 26]. Patients were divided into 2 groups (pCR and non-pCR) according to the pathological response.

### Preparation of RNA and DNA

Frozen specimens were homogenized, and total RNA was extracted using QIAamp^TM^ DNA Mini Kit (QIAGEN Inc., Valencia, CA) and QIAGEN RNeasy^TM^ mini kit (QIAGEN), according to the manufacture’s protocol. Total RNA (200 ng) was reverse transcribed to cDNA using murine leukemia virus reverse transcriptase (Invitrogen Crop., Carlsbad, CA). Our laboratory protocols are deposited in protocols.io (https://dx.doi.org/10.17504/protocols.io.kahcsb6).

### Gene expression analysis using microarray analysis

A human 8 × 60 K whole genome oligo DNA microarray chip (SurePrint G3 Human Gene Expression v3 Microarray Kit, G4851C, Agilent Technologies, Santa Clara, CA) was used for global gene expression analysis, according to the manufacturer’s protocol. Cyanine (Cy)-labeled cRNA was prepared using T7 linear amplification, according to the Agilent Low RNA Input Fluorescent Linear Amplification Manual (Agilent Technologies). Labeled cRNA was fragmented and hybridized to the same oligonucleotide microarray (Agilent Technologies). The fluorescent intensities were determined with an Agilent DNA Microarray Scanner and analyzed as described using Feature Extraction v.10.7.3.1 (Agilent Technologies). Expression levels were converted into log_2_ values and normalized to the median of the entire spot array using GeneSpring^TM^ GX11 (Agilent Technologies). Following normalization, log_2_ fold change (log_2_FC) in gene expression was calculated using Microsoft Excel^®^ 2016 (Microsoft Corp., Redmond, WA), and the formula was as follows:

$${log}_{2}FC=\frac{{log}_{2}(molecular\;expression\;of\;pCR)}{{log}_{2}(molecular\;expression\;of\;Non-pCR)}$$

Further analysis was performed using KeyMolnet.

### Molecular expression analysis using KeyMolnet

The molecular networks and pathways were analyzed using the KeyMolnet Viewer program version 6.1 (KM Data; www.km-data.jp). KeyMolnet, another commercial knowledge base, has manually curated content on 164,000 relationships among human genes and proteins, small molecules, diseases, pathways, and drugs. It includes core content collected from selected review articles with the highest reliability [27].

KeyMolnet automatically provides corresponding molecules as a node on the networks, by importing the list of Entrez Gene ID and signal intensity data [28, 29]. In this study, gene data, for which expressions were significantly different between the pCR group and non-pCR group, were imported into KeyMolnet. Subsequently, the molecular expressions were calculated and the molecules, which were included in the canonical networks of cancer chemotherapy, were isolated as candidate molecules.

### Molecular pathway analysis using KeyMolnet

To identify the relations of the candidate molecules and canonical pathways, pathway analyses were performed. An algorithm that counts the number of overlapping molecular relations between the extracted network and the canonical pathway allows the identification of the canonical pathway showing the most significant contribution to the extracted network. The significance in the similarity between both was scored using the following formula:

$$Score\;(p)=\overset{Min(C,V)}{\underset{x=O}{\sum }}\;f(x)$$

$$\left(f(x)={}_{C}C_{x}\;・\;{}_{T-C}C_{V-x}/{}_{T}C_{V}\right)$$

$$Score=-{log}_{2}\;(Score\;(p))$$

where O = the number of overlapping molecular relations between the extracted network and the canonical pathway, V = the number of molecular relations located in the extracted network, C = the number of molecular relations located in the canonical pathway, T = the number of total molecular relations (approximately 90,000 sets), and X = the sigma variable that defines incidental agreements [29, 30].

This calculation formula contained the hypergeometric distribution, and the score of more than 20 was considered statistically significant.

### Validation study

To validate the feasibility of the expression of candidate genes, a validation study was conducted in another cohort. This was a cohort study of locally advanced esophageal cancer patients treated with NAC-DCF followed by surgery at Oita University Hospital between April and October 2016. Seven cases were enrolled in this study. The cases were divided into the following 2 groups: pCR group and non-pCR group, and we investigated candidate molecular expression using DNA microarray data and KeyMolnet.

### Statistical analysis

Quantitative clinical data are presented as medians and ranges. The difference between groups was assessed using the chi-square test, Fisher’s exact test, or Mann–Whitney *U* test, as appropriate. These analyses were carried out using EZR (Saitama Medical Center, Jichi Medical University, Saitama, Japan version 1.33) [31], which is a graphical user interface for R (version 3.3.1; The R Foundation for Statistical Computing, Vienna, Austria). More precisely, EZR is a modified version of R commander (version 2.3–0) designed to add statistical functions frequently used in biostatistics. A *P*-value < 0.05 was considered statistically significant.

Quantitative gene expression data are presented as means. The difference in gene expression between groups was assessed using Student’s *t*-test and Excel 2016 (Microsoft). A *P*-value < 0.05 was considered statistically significant.

## Results

### Patient characteristics

This study enrolled 32 consecutive cases. Clinicopathological characteristics are shown in Table 1. Of the 32 cases, 9 were included in the pCR group and 23 were included in the non-pCR group, according to the histopathological response grade (S1 Table). There were no differences between the 2 groups in terms of age, sex, cT, cN, cStage, pN, residual tumor, NAC adverse events, procedure, and postoperative complications. However, significant differences were observed in pT (*P* < 0.01) and pStage (*P* < 0.01) between the 2 groups. With regard to NAC, 1 case received only 2 courses, because cancer progression was observed during preoperative treatment. With regard to NAC adverse events, 27 of the 32 cases showed grade 3 or 4 events, according to the CTCAE ver. 4.0 classification. Twenty-four cases showed myelosuppression (6 cases in the pCR group and 18 cases in the non-pCR group). Other events included oral mucositis, appetite loss, and eruption. No mortality cases associated with NAC-DCF and surgery were noted among all the study cases. With regard to postoperative complications, 2 cases in the pCR group and 1 case in the non-pCR group showed grade 3 or 4 events, according to the Clavien-Dindo classification system. Of the 2 cases in the pCR group, 1 had a necrotic bronchus and the other had a diaphragmatic hernia. The 1 case from the non-pCR group had anastomotic leakage.

**Table 1:** Clinicopathological data.

	pCR	Non-pCR	*P*-value
	(n = 9)	(n = 23)	
Age (years); mean ± SD	67 ± 10	64 ± 8	0.454
Sex			0.49
Male	8	22	
Female	1	1	
cT			0.13
1b	2	0	
2	2	5	
3	4	16	
4	1	2	
cN			0.142
0	5	5	
1	2	12	
2	2	6	
3	0	0	
cStage			0.242
IB	2	2	
II	3	4	
III	4	17	
pT			<0.01
0 (CR)	9	0	
1a/1b	0	9	
2	0	4	
3	0	7	
4	0	3	
pN			0.054
0	9	7	
1	0	7	
2	0	5	
3	0	4	
pStage			<0.01
0 (pCR)	9	0	
IA/IB	0	5	
II	0	8	
III	0	10	
Residual tumor			0.303
R0	9	19	
R1/R2	0	4	
Histopathological response grade			<0.01
0	0	1	
1a/1b	0	14	
2	0	8	
3	9	0	
Accomplishment of NAC			1.00
Complete	9	22	
Incomplete	0	1	
Adverse events of NAC^a^			0.604
Absent	2	3	
Present	7	20	
Procedure			0.541
Subtotal esophagectomy	9	20	
Others	0	3	
Postoperative complications^b^			0.184
Absent	7	22	
Present	2	1	

^a^Grade 3/4 according to CTCAE ver. 4.0

^b^Grade 3/4 according to the Clavien-Dindo classification system

TNM stage was classified according to UICC 7th edition

### Molecular expression to predict pCR after NAC-DCF for esophageal cancer

**Fig 1:**
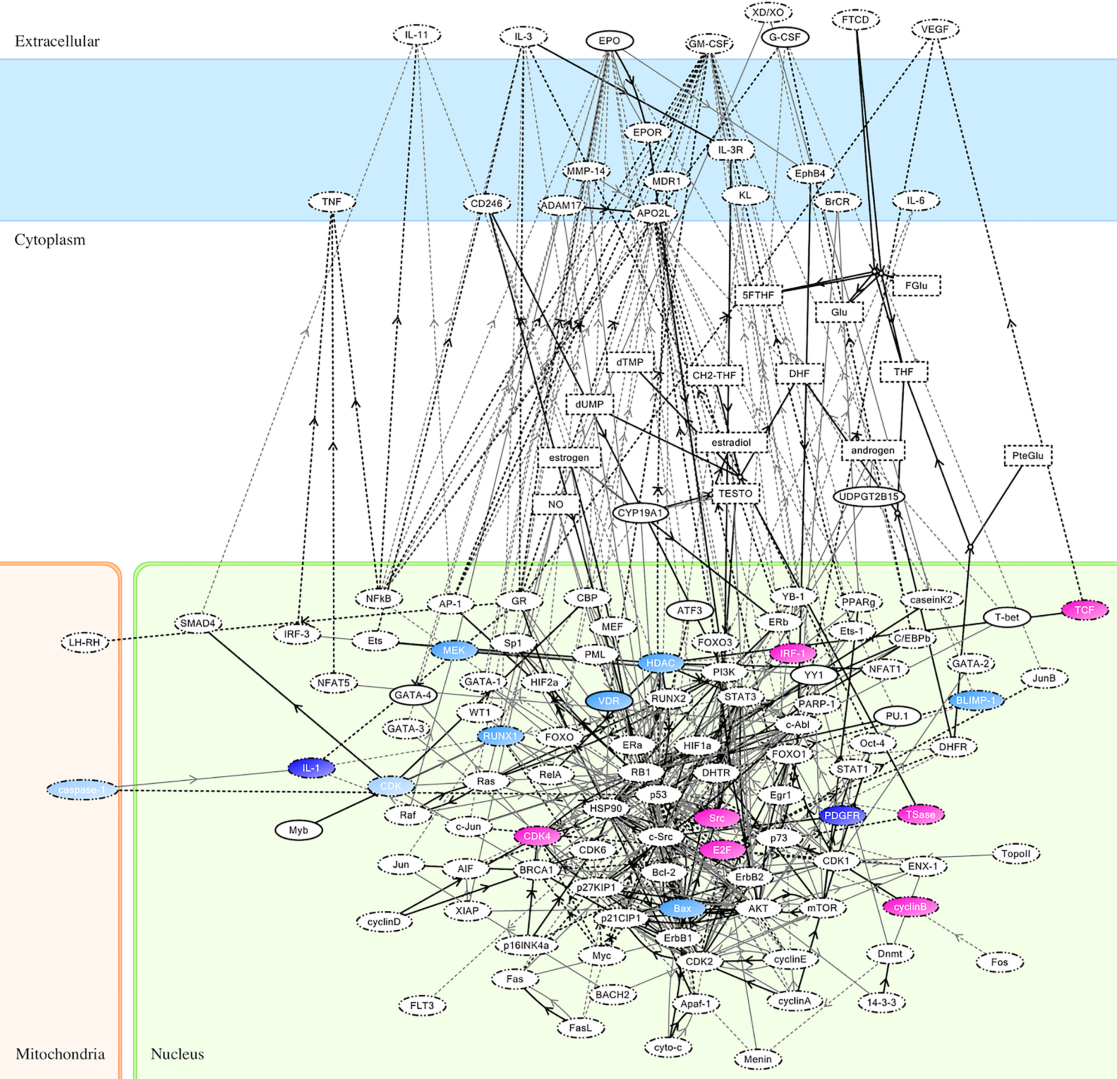
Color mapping of molecular expressions on KeyMolnet. Seventeen molecules were included in KeyMolnet. Red nodes indicate higher expression in the pCR group. Blue nodes indicate lower expression in the pCR group. The color shades are correlated with the expression levels of the molecules.

Significant differences in the gene expressions of 1,891 genes were observed between the pCR and non-pCR groups (S2 Table). On importing data of the 1,891 gene expressions into KeyMolnet and evaluating the molecular expressions associated with the canonical networks of cancer chemotherapy, 17 molecules were isolated as candidate molecules. They included transcription factor E2F (E2F), T-cell-specific transcription factor (TCF), Src (known as “proto-oncogene tyrosine-protein kinase of sarcoma”), interferon regulatory factor 1 (IRF-1), thymidylate synthase (TSase), cyclin B, cyclin-dependent kinase (CDK) 4, CDK, caspase-1, vitamin D receptor (VDR), histone deacetylase (HDAC), MAPK/ERK kinase (MEK), bcl-2-associated X protein (Bax), runt-related transcription factor 1 (RUNX1), PR domain zinc finger protein 1 (BLIMP-1), platelet-derived growth factor receptor (PDGFR), and interleukin 1 (IL-1). The color mapping of these 17 molecules on KeyMolnet of canonical molecular networks associated with cancer chemotherapy are displayed in Fig 1. A red node indicated higher expression in the pCR group than in the non-pCR group, while a blue node indicated lower expression in the pCR group than in the non-pCR group. The expressions of the 17 molecules are summarized in Table 2. The putative molecular functions were referred to UniProt (http://www.uniprot.org).

**Table 2:** Fold change (FC) of the 17 identified molecules.

Molecule	Putative molecular function	log_2_FC	*P*-value
E2F	Transcription activator associated with cell cycle regulation or DNA replication.	0.767	<0.001
TCF	Transcription activator involved in T-cell lymphocyte differentiation.	0.761	0.025
Src	Non-receptor protein tyrosine kinase that activates many different classes of cellular receptors.	0.755	0.011
IRF-1	Transcriptional regulator that displays functional diversity in the regulation of cellular responses.	0.675	0.01
TSase	Contributes to the de novo mitochondrial thymidylate biosynthesis pathway.	0.655	0.006
Cyclin B	Essential for the control of the cell cycle at the G2/M (mitosis) transition.	0.604	0.027
CDK4	Ser/Thr-kinase component that phosphorylates and inhibits members of the RB protein and regulates the cell cycle during G1/S transition.	0.523	0.03
CDK	Appears to play multiple roles in cell cycle progression, cytokinesis, and apoptosis.	−0.042	0.03
Caspase-1	Thiol protease involved in a variety of inflammatory processes.	−0.231	0.019
VDR	Transcription factor that mediates the action of vitamin D3.	−0.529	0.031
HDAC	Responsible for the deacetylation of lysine residues on the N-terminal part of core histones.	−0.565	0.007
MEK	Dual specificity protein kinase of the MAP kinase signal transduction pathway.	−0.603	0.034
Bax	Accelerates programmed cell death by binding to and antagonizing the apoptosis repressor.	−0.609	0.015
RUNX1	Transcriptional factor associated with the differentiation of the hematopoietic system.	−0.760	0.014
BLIMP-1	Transcription factor that mediates a transcriptional program in various immune tissue-resident lymphocyte T-cell types.	−0.810	0.007
PDGFR	Tyrosine-protein kinase associated with the regulation of embryonic development, cell proliferation, survival, and chemotaxis.	−1.147	0.002
IL-1	Involved in the inflammatory response and stimulates the release of prostaglandin and collagenase from synovial cells.	−1.173	0.024

### Molecular pathway associated with pCR after NAC-DCF for esophageal cancer

On analyzing the relationships between canonical pathways and extracted molecules using the score of hypergeometric distribution, 47 pathways scored more than 20 and were considered to be significantly associated with the extracted molecules (S3 Table). The 3 pathways with the highest scores were transcriptional regulation by SMAD (score 96.054), retinoblastoma protein (RB)/E2F (score 77.067), and signal transducer and activator of transcription (STAT) (score 76.942).

### Validation study of candidate molecules to predict pCR after NAC-DCF for esophageal cancer

**Fig 2:**
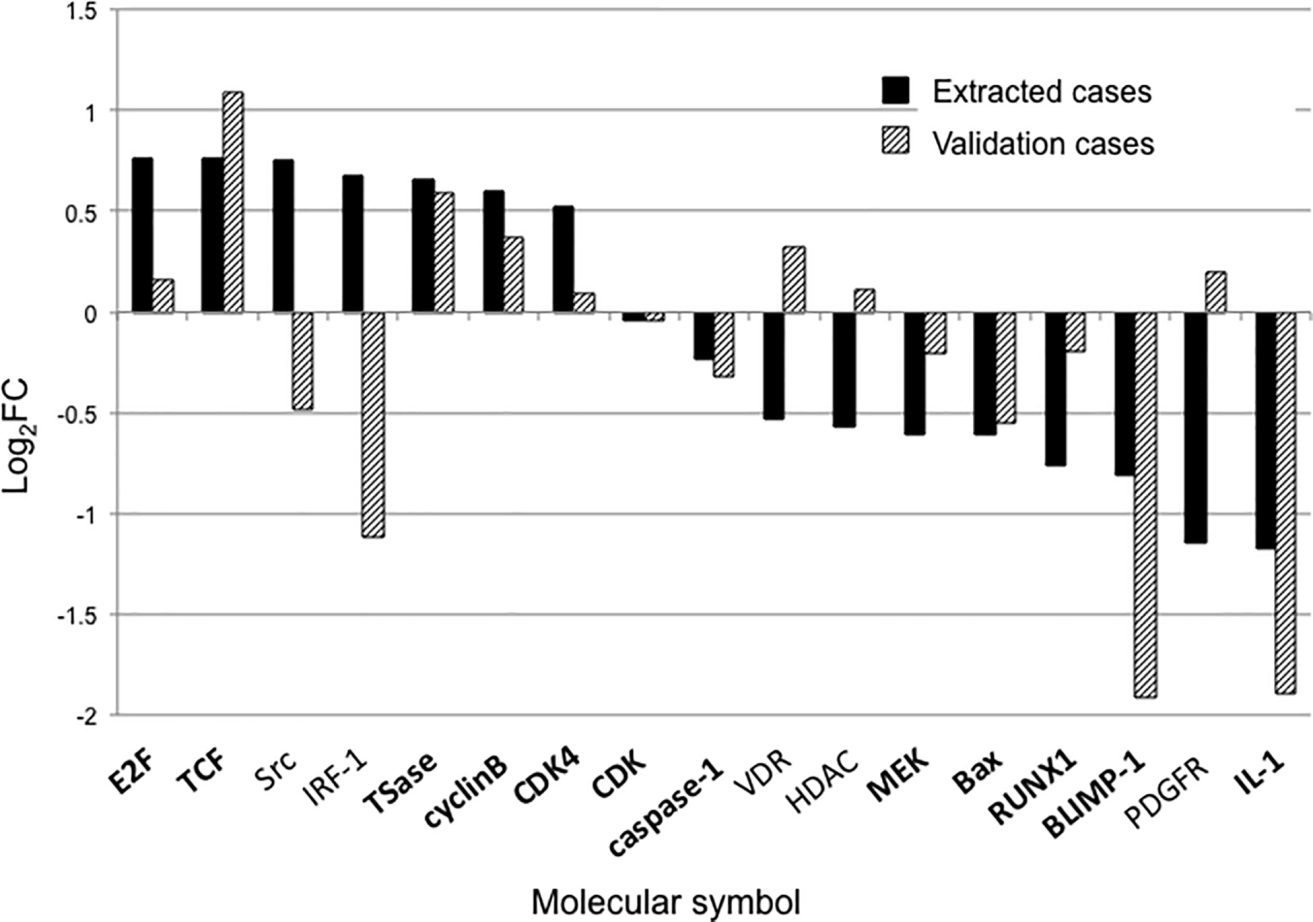
Comparison of candidate molecular expressions between extracted cases and validation cases. Of the 17 molecules, 12 (71%) (bold) matched the trends of molecular expression.

Of the 7 validation cases, 1 case was classified in the pCR group and 6 cases were classified in the non-pCR group (S1 Table). The clinicopathological characteristics of the validation cases are summarized in Table 3. The comparison of molecular expressions between extracted cases and validation cases is presented in Fig 2 (S4 Table). Of the 17 molecules, 12 (71%) matched the trends of molecular expression, including E2F, TCF, TSase, cyclin B, CDK4, CDK, caspase-1, MEK, Bax, RUNX1, BLIMP-1, and IL-1.

**Table 3:** Clinicopathological data of the validation study.

Case no.	Histopathological response grade	Age (years)	Sex	cStage	pStage	Accomplishment of NAC
1	1a	78	Male	IB	IIA	Complete
2	2	63	Male	IIIA	IA	Complete
3	1a	67	Male	IIIA	IIIA	Complete
4	2	68	Male	IIB	X*	Complete
5	1a	80	Male	IIA	IIIA	Complete
6	2	66	Male	IIIA	IA	Complete
7	3	62	Male	IIIC	0 (pCR)	Complete

*Case no. 4 was diagnosed with pStage X, because the primary lesion was pCR, although viable cancer cells were observed in the lymph node lesion.

## Discussion

We observed a high pCR rate (28%) on treatment with NAC-DCF for locally advanced esophageal cancer. We explored the predictors of pCR using biopsy specimens of locally advanced esophageal cancer patients treated with NAC-DCF. In the present study, 17 molecules were identified as predictors of pCR after NAC-DCF for locally advanced esophageal cancer, using DNA microarray and KeyMolnet. The validation study indicated that 12 of 17 molecules (71%) matched the trends of molecular expression. These 17 molecules are expected to be a predictor set of pCR after NAC-DCF for locally advanced esophageal cancer.

So far, several predictors of chemosensitivity in esophageal cancer have been reported; however, no biomarkers have been developed for clinical application in esophageal cancer. Our identified molecules involved several biomarkers of chemosensitivity or therapeutic target in esophageal cancer. TSase is commonly known as a target molecule of 5-FU in malignant tumors, and it was reported that the expression of *TSase* was correlated with 5-FU sensitivity in esophageal cancer [32]. Accordingly, our data was considered credible, because well-known molecules, such as TSase, were included in our set of candidate molecules. Additionally, it was reported that the expression of E2F1 was associated with prognosis in esophageal cancer, and it might be a candidate target molecule for chemosensitivity of esophageal cancer [33]. It was reported that the expression of IRF-1 inhibited the growth of esophageal cancer cells and that IRF-1 had a potential effect as a tumor suppressor in esophageal cancer [34]. However, we could not identify reports investigating chemosensitivity with regard to TCF, Src, caspase-1, VDR, BLIMP-1, PDGFR, and IL-1 in esophageal cancer. Accordingly, we consider that our identified 17 molecules are possible novel biomarkers of chemosensitivity in esophageal cancer. A previous study showed that multiple factors are involved in drug response mechanisms [13]; therefore, we hypothesized that our identified 17 molecules might intricately interact. Therefore, we analyzed the relationships between canonical pathways and extracted molecules using KeyMolnet and determined the relationship score based on hypergeometric distribution.

According to the pathway analysis results, 17 molecules were considered to be associated with 47 pathways, and the following 3 pathways had the highest scores: SMAD, RB/E2F, and STAT. SMAD is a TCF in the transforming growth factor-β (TGF-β) signaling pathway (TGF-β/SMAD signaling pathway), which promotes cell proliferation. SMAD is associated with carcinogenesis, cancer proliferation, and invasion. Several studies have reported that 5-FU inhibited this pathway and that cell proliferation was inhibited by 5-FU in TGF-β negative cases [35, 36]. The RB/E2F pathway is critical for regulating the initiation of DNA replication. It is known that the control of this pathway is disrupted in virtually all human cancers. A previous study reported that inhibition of the RB/E2F pathway suppressed tumor growth and increased the effect of gemcitabine in pancreatic cancer [37]. On the other hand, several reports have indicated that inhibition of the RB/E2F pathway decreased the effect of CDDP in lung cancer and breast cancer [38, 39] and decreased the effect of paclitaxel in lung cancer [40]. STAT is a TCF activated by JAK in the JAK/STAT pathway. Activation of this pathway is associated with T-cell activation and tumor immunity. It has been reported that inactivation of the JAK/STAT pathway inhibited tumor proliferation in esophageal cancer [41]. However, it has been reported that activation of the JAK/STAT pathway increased CDDP sensitivity in head and neck squamous cell carcinoma [42]. On investigating the molecular associations of these pathways, we found that CDK4, HDAC, RUNX1, and VDR were associated with SMAD through the TGF-β/SMAD signaling pathway. Additionally, CDK4, E2F, TSase, and HDAC were associated with the RB/E2F pathway. Moreover, Src and IRF-1 were associated with the JAK/STAT pathway. These pathways were mainly associated with chemosensitivity with regard to pCR in our study.

Several reports revealed the survival benefit in pCR cases after neoadjuvant therapy for esophageal cancer and suggested the necessity of a useful predictor of pCR [10, 11]. Furthermore, studies have reported some examination methods and biomarkers to predict pCR after neoadjuvant therapy for esophageal cancer, although no predictors of pCR have been developed for clinical application in esophageal cancer [43–45]. A previous study found no significant differences in the oncological outcomes of clinical complete response (cCR) cases between a no treatment group and a radical surgery group after neoadjuvant chemoradiotherapy or radiotherapy for advanced esophageal cancer [46], and the data suggested a “watch and wait” policy for cases with cCR in order to avoid the morbidity associated with radical surgery. We expect that our identified 17-molecule set will contribute to the prediction of pCR and will help with the “watch and wait” policy in cases of esophageal cancer. We intend to further evaluate the feasibility of the predictive value of our identified predictor set.

The present study has several limitations. First, conception of heterogeneity among cancer tissues should be considered. Therefore, we ensured that biopsy specimens were uniformly collected from an elevated part at the proximal side of the tumor. Second, the expressions of candidate molecules were not confirmed using PCR, western blotting, and immunostaining. Therefore, we intend to perform further research on these molecules. Third, the present study has a small sample size and is retrospective in design, including the validation cohort. The validation method only compared the trends of molecular expression between pCR and non-pCR cases, and did not evaluate the predictive values of the candidate molecules. Next, we intend to collect more samples and evaluate the predictive values. The biological functions should also be investigated. Fourth, the present retrospective data were likely influenced by selection bias, which did not merely include difficult lesions for resection, such as large tumors. Hence, the present observations require confirmation in prospective studies.

In conclusion, a 17-molecule set that can predict pCR after NAC-DCF for locally advanced esophageal cancer was identified using pretreatment biopsy samples. We intend to perform further research on these molecules and their pathways, and conduct a prospective study to evaluate the feasibility of the predictive value of this molecule set.

## Supporting information

S1 Table Case classification of test and validation cohorts. Histopathologic response grade and response classification are summarized in this table.
(XLSX)

S2 Table Expression of 1891 genes. The expression of 1891 genes, which were observed significant differences between pCR group and non-pCR group, are shown in this table.
(XLSX)

S3 Table Pathway score. 47 pathways, which scored more than 20 using the score of hypergeometric distribution, are listed in this table.
(XLSX)

S4 Table Molecular expression of validation cases. The molecular expression of validation cases were summarized in this table.
(XLSX)

## References

[1] Medical Research Council Oesophageal Cancer Working Party. Surgical resection with or without preoperative chemotherapy in oesophageal cancer: a randomised controlled trial. Lancet. 2002;359:1727–1733. doi: 10.1016/S0140-6736(02)08651-8 12049861 10.1016/S0140-6736(02)08651-8

[2] Tanaka E, Okabe H, Tsunoda S, Obama K, Kan T, Kadokawa Y, et al Feasibility of thoracoscopic esophagectomy after neoadjuvant chemotherapy. Asian J Endosc Surg. 2012;5:111–117. doi: 10.1111/j.1758-5910.2012.00131.x 22776501 10.1111/j.1758-5910.2012.00131.x

[3] Cunningham D, Allum WH, Stenning SP, Thompson JN, Van de Velde CJ, Nicolson M, et al Perioperative chemotherapy versus surgery alone for resectable gastroesophageal cancer. N Engl J Med. 2006;355:11–20. doi: 10.1056/NEJMoa055531 16822992 10.1056/NEJMoa055531

[4] Ychou M, Boige V, Pignon JP, Conroy T, Bouche O, Lebreton G, et al Perioperative chemotherapy compared with surgery alone for resectable gastroesophageal adenocarcinoma: an FNCLCC and FFCD multicenter phase III trial. J Clin Oncol. 2011;29:1715–1721. doi: 10.1200/JCO.2010.33.0597 21444866 10.1200/JCO.2010.33.0597

[5] Kitagawa Y, Idani H, Inoue H, Udagawa H, Uyama I, Osugi H, et al Gastroenterological surgery: esophagus. Asian J Endosc Surg. 2015;8:114–124. doi: 10.1111/ases.12185 25913582 10.1111/ases.12185

[6] Ando N, Kato H, Igaki H, Shinoda M, Ozawa S, Shimizu H, et al A randomized trial comparing postoperative adjuvant chemotherapy with cisplatin and 5-fluorouracil versus preoperative chemotherapy for localized advanced squamous cell carcinoma of the thoracic esophagus (JCOG9907). Ann Surg Oncol. 2012;19:68–74. doi: 10.1245/s10434-011-2049-9 21879261 10.1245/s10434-011-2049-9

[7] Hara H, Tahara M, Daiko H, Kato K, Igaki H, Kadowaki S, et al Phase II feasibility study of preoperative chemotherapy with docetaxel, cisplatin, and fluorouracil for esophageal squamous cell carcinoma. Cancer Sci. 2013;104:1455–1460. doi: 10.1111/cas.12274 23991649 10.1111/cas.12274 PMC7654256

[8] Ojima T, Nakamori M, Nakamura M, Katsuda M, Hayata K, Kato T, et al Neoadjuvant Chemotherapy with Divided-dose Docetaxel, Cisplatin and Fluorouracil for Patients with Squamous Cell Carcinoma of the Esophagus. Anticancer Res. 2016;36:829–834. 26851048

[9] Nomura M, Oze I, Abe T, Komori A, Narita Y, Masuishi T, et al Impact of docetaxel in addition to cisplatin and fluorouracil as neoadjuvant treatment for resectable stage III or T3 esophageal cancer: a propensity score-matched analysis. Cancer Chemother Pharmacol. 2015;76:357–363. doi: 10.1007/s00280-015-2806-8 26092324 10.1007/s00280-015-2806-8

[10] Watanabe M, Baba Y, Yoshida N, Ishimoto T, Nagai Y, Iwatsuki M, et al Outcomes of preoperative chemotherapy with docetaxel, cisplatin, and 5-fluorouracil followed by esophagectomy in patients with resectable node-positive esophageal cancer. Ann Surg Oncol. 2014;21:2838–2844. doi: 10.1245/s10434-014-3684-8 24715216 10.1245/s10434-014-3684-8

[11] Molena D, Sun HH, Badr AS, Mungo B, Sarkaria IS, Adusumilli PS, et al Clinical tools do not predict pathological complete response in patients with esophageal squamous cell cancer treated with definitive chemoradiotherapy. Dis Esophagus. 2014;27:355–359. doi: 10.1111/dote.12126 24033404 10.1111/dote.12126

[12] Evans WE, Relling MV. Pharmacogenomics: translating functional genomics into rational therapeutics. Science. 1999;286:487–491. 10521338 10.1126/science.286.5439.487

[13] Fumoto S, Shimokuni T, Tanimoto K, Hiyama K, Otani K, Ohtaki M, et al Selection of a novel drug-response predictor in esophageal cancer: a novel screening method using microarray and identification of IFITM1 as a potent marker gene of CDDP response. Int J Oncol. 2008;32:413–423. 18202764

[14] Gao Y, Zhu J, Zhang X, Wu Q, Jiang S, Liu Y, et al BRCA1 mRNA expression as a predictive and prognostic marker in advanced esophageal squamous cell carcinoma treated with cisplatin- or docetaxel-based chemotherapy/chemoradiotherapy. PLoS One. 2013;8:e52589 doi: 10.1371/journal.pone.0052589 23326344 10.1371/journal.pone.0052589 PMC3541365

[15] Li L, Xu N, Fan N, Meng Q, Luo W, Lv L, et al Upregulated KLK10 inhibits esophageal cancer proliferation and enhances cisplatin sensitivity in vitro. Oncol Rep. 2015;34:2325–2332. doi: 10.3892/or.2015.4211 26479703 10.3892/or.2015.4211

[16] Harpole DH Jr., Moore MB, Herndon JE 2nd, Aloia T, D'Amico TA, Sporn T, et al The prognostic value of molecular marker analysis in patients treated with trimodality therapy for esophageal cancer. Clin Cancer Res. 2001;7:562–569. 11297249

[17] Joshi MB, Shirota Y, Danenberg KD, Conlon DH, Salonga DS, Herndon JE 2nd, et al High gene expression of TS1, GSTP1, and ERCC1 are risk factors for survival in patients treated with trimodality therapy for esophageal cancer. Clin Cancer Res. 2005;11:2215–2221. doi: 10.1158/1078-0432.CCR-04-1387 15788669 10.1158/1078-0432.CCR-04-1387

[18] Nakano J, Huang C, Liu D, Masuya D, Nakashima T, Yokomise H, et al Evaluations of biomarkers associated with 5-FU sensitivity for non-small-cell lung cancer patients postoperatively treated with UFT. Br J Cancer. 2006;95:607–615. doi: 10.1038/sj.bjc.6603297 16880781 10.1038/sj.bjc.6603297 PMC2360692

[19] Mori R, Ishiguro H, Kuwabara Y, Kimura M, Mitsui A, Tomoda K, et al Targeting beta1 integrin restores sensitivity to docetaxel of esophageal squamous cell carcinoma. Oncol Rep. 2008;20:1345–1351. 19020712

[20] Hiyoshi Y, Yoshida N, Watanabe M, Kurashige J, Baba Y, Sakamoto Y, et al The Presence of Serum p53 Antibody Predicts the Pathological Tumor Response to Neoadjuvant Chemotherapy with Docetaxel, Cisplatin and Fluorouracil (DCF) in Esophageal Squamous Cell Carcinoma. World J Surg. 2017;41:480–486. doi: 10.1007/s00268-016-3649-0 27637603 10.1007/s00268-016-3649-0

[21] McLeod HL, Evans WE. Pharmacogenomics: unlocking the human genome for better drug therapy. Annu Rev Pharmacol Toxicol. 2001;41:101–121. doi: 10.1146/annurev.pharmtox.41.1.101 11264452 10.1146/annurev.pharmtox.41.1.101

[22] Staunton JE, Slonim DK, Coller HA, Tamayo P, Angelo MJ, Park J, et al Chemosensitivity prediction by transcriptional profiling. Proc Natl Acad Sci U S A. 2001;98:10787–10792. doi: 10.1073/pnas.191368598 11553813 10.1073/pnas.191368598 PMC58553

[23] Sun Z, Yang P. Gene expression profiling on lung cancer outcome prediction: present clinical value and future premise. Cancer Epidemiol Biomarkers Prev. 2006;15:2063–2068. doi: 10.1158/1055-9965.EPI-06-0505 17119029 10.1158/1055-9965.EPI-06-0505

[24] Empuku S, Nakajima K, Akagi T, Kaneko K, Hijiya N, Etoh T, et al An 80-gene set to predict response to preoperative chemoradiotherapy for rectal cancer by principle component analysis. Mol Clin Oncol. 2016;4:733–739. doi: 10.3892/mco.2016.806 27123272 10.3892/mco.2016.806 PMC4840568

[25] Society JE. Japanese Classification of Esophageal Cancer, 11th Edition: part I. Esophagus. 2017;14:1–36. doi: 10.1007/s10388-016-0551-7 28111535 10.1007/s10388-016-0551-7 PMC5222932

[26] Society JE. Japanese Classification of Esophageal Cancer, 11th Edition: part II and III. Esophagus. 2017;14:37–65. doi: 10.1007/s10388-016-0556-2 28111536 10.1007/s10388-016-0556-2 PMC5222925

[27] Satoh J, Asahina N, Kitano S, Kino Y. A Comprehensive Profile of ChIP-Seq-Based Olig2 Target Genes in Motor Neuron Progenitor Cells Suggests the Possible Involvement of Olig2 in the Pathogenesis of Amyotrophic Lateral Sclerosis. J Cent Nerv Syst Dis. 2015;7:1–14. doi: 10.4137/JCNSD.S23210 26023283 10.4137/JCNSD.S23210 PMC4437538

[28] Kuzuhara T, Suganuma M, Kurusu M, Fujiki H. Helicobacter pylori-secreting protein Tipalpha is a potent inducer of chemokine gene expressions in stomach cancer cells. J Cancer Res Clin Oncol. 2007;133:287–296. doi: 10.1007/s00432-006-0169-6 17393199 10.1007/s00432-006-0169-6 PMC12160870

[29] Satoh J, Illes Z, Peterfalvi A, Tabunoki H, Rozsa C, Yamamura T. Aberrant transcriptional regulatory network in T cells of multiple sclerosis. Neurosci Lett. 2007;422:30–33. doi: 10.1016/j.neulet.2007.05.056 17629622 10.1016/j.neulet.2007.05.056

[30] Satoh J, Obayashi S, Misawa T, Sumiyoshi K, Oosumi K, Tabunoki H. Protein microarray analysis identifies human cellular prion protein interactors. Neuropathol Appl Neurobiol. 2009;35:16–35. doi: 10.1111/j.1365-2990.2008.00947.x 18482256 10.1111/j.1365-2990.2008.00947.x

[31] Kanda Y. Investigation of the freely available easy-to-use software 'EZR' for medical statistics. Bone Marrow Transplant. 2013;48:452–458. doi: 10.1038/bmt.2012.244 23208313 10.1038/bmt.2012.244 PMC3590441

[32] Ando T, Ishiguro H, Kuwabara Y, Kimura M, Mitsui A, Sugito N, et al Relationship between expression of 5-fluorouracil metabolic enzymes and 5-fluorouracil sensitivity in esophageal carcinoma cell lines. Dis Esophagus. 2008;21:15–20. doi: 10.1111/j.1442-2050.2007.00700.x 18197934 10.1111/j.1442-2050.2007.00700.x

[33] Wang W, Shen L, Sun Y, Dong B, Chen K. Role of E2F-1 and its involving pathway in esophageal squamous cell carcinoma. Thorac Cancer. 2014;5:139–148. doi: 10.1111/1759-7714.12061 26766991 10.1111/1759-7714.12061 PMC4704324

[34] Wang Y, Liu DP, Chen PP, Koeffler HP, Tong XJ, Xie D. Involvement of IFN regulatory factor (IRF)-1 and IRF-2 in the formation and progression of human esophageal cancers. Cancer Res. 2007;67:2535–2543. doi: 10.1158/0008-5472.CAN-06-3530 17363571 10.1158/0008-5472.CAN-06-3530

[35] Romano G, Santi L, Bianco MR, Giuffre MR, Pettinato M, Bugarin C, et al The TGF-beta pathway is activated by 5-fluorouracil treatment in drug resistant colorectal carcinoma cells. Oncotarget. 2016;7:22077–22091. doi: 10.18632/oncotarget.7895 26956045 10.18632/oncotarget.7895 PMC5008345

[36] Wendling J, Marchand A, Mauviel A, Verrecchia F. 5-fluorouracil blocks transforming growth factor-beta-induced alpha 2 type I collagen gene (COL1A2) expression in human fibroblasts via c-Jun NH2-terminal kinase/activator protein-1 activation. Mol Pharmacol. 2003;64:707–713. doi: 10.1124/mol.64.3.707 12920208 10.1124/mol.64.3.707

[37] Batchu RB, Gruzdyn OV, Bryant CS, Qazi AM, Kumar S, Chamala S, et al Ritonavir-Mediated Induction of Apoptosis in Pancreatic Cancer Occurs via the RB/E2F-1 and AKT Pathways. Pharmaceuticals (Basel). 2014;7:46–57. 24451403 10.3390/ph7010046 PMC3915194

[38] Reed MF, Zagorski WA, Knudsen ES. RB activity alters checkpoint response and chemosensitivity in lung cancer lines. J Surg Res. 2007;142:364–372. doi: 10.1016/j.jss.2007.03.038 17640669 10.1016/j.jss.2007.03.038 PMC2734970

[39] Bosco EE, Wang Y, Xu H, Zilfou JT, Knudsen KE, Aronow BJ, et al The retinoblastoma tumor suppressor modifies the therapeutic response of breast cancer. J Clin Invest. 2007;117:218–228. doi: 10.1172/JCI28803 17160137 10.1172/JCI28803 PMC1679964

[40] Kurtyka CA, Chen L, Cress WD. E2F inhibition synergizes with paclitaxel in lung cancer cell lines. PLoS One. 2014;9:e96357 doi: 10.1371/journal.pone.0096357 24831239 10.1371/journal.pone.0096357 PMC4022639

[41] Liu JR, Wu WJ, Liu SX, Zuo LF, Wang Y, Yang JZ, et al Nimesulide inhibits the growth of human esophageal carcinoma cells by inactivating the JAK2/STAT3 pathway. Pathol Res Pract. 2015;211:426–434. doi: 10.1016/j.prp.2015.01.007 25724470 10.1016/j.prp.2015.01.007

[42] Hato SV, Figdor CG, Takahashi S, Pen AE, Halilovic A, Bol KF, et al Direct inhibition of STAT signaling by platinum drugs contributes to their anti-cancer activity. Oncotarget. 2017. 10.18632/oncotarget.17661 PMC5589592 28903353

[43] Huang RW, Chao YK, Wen YW, Chang HK, Tseng CK, Chan SC, et al Predictors of pathological complete response to neoadjuvant chemoradiotherapy for esophageal squamous cell carcinoma. World J Surg Oncol. 2014;12:170 doi: 10.1186/1477-7819-12-170 24885430 10.1186/1477-7819-12-170 PMC4050419

[44] Smit JK, Faber H, Niemantsverdriet M, Baanstra M, Bussink J, Hollema H, et al Prediction of response to radiotherapy in the treatment of esophageal cancer using stem cell markers. Radiother Oncol. 2013;107:434–441. doi: 10.1016/j.radonc.2013.03.027 23684587 10.1016/j.radonc.2013.03.027

[45] Sohda M, Honjyo H, Hara K, Ozawa D, Suzuki S, Tanaka N, et al L-[3-18F]-alpha-methyltyrosine accumulation as a definitive chemoradiotherapy response predictor in patients with esophageal cancer. Anticancer Res. 2014;34:909–913. 24511031

[46] Adenis A, Tresch E, Dewas S, Romano O, Messager M, Amela E, et al Clinical complete responders to definite chemoradiation or radiation therapy for oesophageal cancer: predictors of outcome. BMC Cancer. 2013;13:413 doi: 10.1186/1471-2407-13-413 24010566 10.1186/1471-2407-13-413 PMC3844443

